# Serum biomarker-based risk model construction for primary Sjögren’s syndrome with interstitial lung disease

**DOI:** 10.3389/fmolb.2024.1448946

**Published:** 2024-08-21

**Authors:** Xiaoli Liu, Xia Zhang, Juan Shi, Shiqing Li, Xiuzhi Zhang, Huiling Wang

**Affiliations:** ^1^ Department of Clinical Laboratory, Henan Provincial People’s Hospital, People’s Hospital of Zhengzhou University, Zhengzhou, China; ^2^ Department of Pathology, Henan Medical College, Zhengzhou, China; ^3^ Department of Ophthalmology, Henan Provincial People’s Hospital, People’s Hospital of Zhengzhou University, Zhengzhou, China

**Keywords:** serum biomarkers, cytokines, primary Sjögren’s syndrome, interstitial lung disease, immune response

## Abstract

**Background:**

Cytokine network disturbances in primary Sjögren’s syndrome (pSS) have been reported in many studies. However, their functions in patients with primary Sjögren’s syndrome and interstitial lung disease (pSS-ILD) is controversial. In this study, we aim to investigate the associations of immunological characteristics and cytokine profiles with pSS-ILD pathogenesis and explore their predictive values for pSS progression.

**Methods:**

A total of 256 patients initially diagnosed with pSS at Henan Provincial People’s Hospital were enrolled. After excluding the patients previously diagnosed with various serious acute and chronic respiratory system diseases and cases with other connective tissue diseases or congenital heart diseases, 94 pSS patients were included for further analysis, including 40 patients with ILD (pSS-ILD) and 54 patients without ILD (pSS-N-ILD). For comparison, 41 age- and sex-matched healthy individuals were included as normal controls. Their clinical symptoms and serological data including cyclic citrullinated peptide (CCP) antibody (anti-CCP), antinuclear antibody (ANA), anti-Ro52, anti-SSA, anti-SSB, C-reactive protein, IgG, IgM, IgA, C3, C4, and 10 cytokines and chemokines were obtained. Wilcoxon test, chi-square test, Spearman correlation analysis, and logistics regression analysis were performed.

**Results:**

Higher positive rates of anti-SSB and higher incidence of dry cough, dyspnea, and arthrosis symptoms were shown in pSS-ILD patients than in the pSS-N-ILD cases. Anti-CCP antibodies and cytokines (IL-1β, TNFα, IL-6, IL-5, IL-12p70, and IL-17) were higher, while C3 was lower in pSS-ILD patients than in pSS-N-ILD cases. Significant negative correlations of IgG with C3 and C4 and positive correlations of IL-12p70 and IL-17 with IL-6 were only shown in pSS-ILD patients. The anti-CCP antibody was positively correlated with IL-5 in pSS-ILD patients, but not in pSS-N-ILD cases. Multi-variable logistics regression analysis revealed the combination of anti-CCP, IL-17, IL-12p70, and IL-5 was effective in predicting the status of pSS-ILD in the pSS cases.

**Conclusion:**

There were significant differences in serum marker levels between pSS-ILD and pSS-N-ILD cases. The combination of anti-CCP, IL-17, IL-12p70, and IL-5 might be a potential risk predictor for pSS-ILD occurrence. The cytokines might be involved in the development and progression of pSS-ILD. These results would provide new therapeutic targets for pSS-ILD treatment.

## Background

Primary Sjögren’s syndrome (pSS) is a systemic autoimmune disease characterized by impaired functions of exocrine glands and the involvement of multiple organs (Luppi et al., 2020a). It is typically associated with the presence of antinuclear antibodies, including anti-SSA, anti-Ro52, and anti-SSB. The extra-glandular involvement determines the prognosis of the patients. The lung is the most commonly involved extra-glandular organ and the source of many symptoms (Mestre-Torres and Solans-Laque, 2022). Interstitial lung disease (ILD) is the main manifestation of lung dysfunctions in pSS patients. It is considered to be the most frequent and serious pulmonary complication in pSS, with a prevalence of approximately 20%, and results in significant morbidity and mortality (Luppi et al., 2020a; Gao et al., 2021; He et al., 2022; Kawada, 2022). Historically, ILD was described as a late manifestation of pSS. However, a high variability of the time of onset of pSS associated with ILD has been observed recently. In fact, from 10% to 51% of the patients can develop ILD years before the onset of pSS (Sambataro et al., 2020).

While the underlying causes of pSS-ILD are not completely clear, it is believed that the cytokines from T and B cells are crucial in triggering and advancing the inflammatory processes (Zhao et al., 2013). pSS patients show an increase in Th17 cells and their characteristic cytokine IL-17, which triggers tissue injury and stimulates responses from autoreactive B cells (Pontarini et al., 2018; Verstappen et al., 2018). IL-12 can affect the production and secretion of IFN-γ (Fogel et al., 2018). IL-5 plays important roles in regulating the differentiation and activation of B cells (Ehrens et al., 2022). It has been noted that Th1 cells generate increased amounts of IFN-γ and TNF-α, which not only lead to the impairment of epithelial cells and glandular function but also stimulate the activity of other immune cells, with a particular impact on B cells (Ewert et al., 2010; Maehara et al., 2012; Ríos-Ríos et al., 2020). TNF-α is a key pro-inflammatory cytokine with a complex involvement in the development of fibrosis (Ríos-Ríos et al., 2020). Sustained activation of TNF-α can initiate pathological fibrosis, identified by an excessive buildup of extracellular matrix (ECM) elements, such as collagen. This excessive accumulation of the ECM disrupts tissue integrity and diminishes organ performance, a defining attribute of fibrotic disorders (Wang et al., 2020). The crucial associations of TGF-β with ILD have been reported in many studies (Godoy et al., 2024; Huang et al., 2024; Xiao et al., 2024). Noticeably, TNF-α can also collaborate with TGF-β to enhance the fibrotic process (Yuan, 2024).

Although the disturbance of cytokine network and immune response have been reported in pSS (Rizzo et al., 2020), the pathogenesis of pSS-ILD is still poorly understood. In recent years, the dysregulations of the cytokines in this disease have attracted considerable attention. It was speculated that excessive production of cytokines might contribute to the progression of pSS to such conditions as ILD, arthralgia, and cancer (Longhino et al., 2023). Recently, relevant reports have found that cytokines may be related to lung inflammation and lead to ILD (Shi et al., 2022). It was reported that patients with pSS-associated ILD presented clinical and serological heterogeneity, which could be influenced by the microenvironment of cytokines (Shi et al., 2020; Weng et al., 2022). However, there are few reports on cytokine profiles in pSS-associated ILD. In this study, we investigated the associations of clinical symptoms, immunological characteristics, and cytokine profiles with pSS to identify clinical risk factors and potential serum biomarkers of discriminating pSS patients with and without ILD (named pSS-ILD and pSS-N-ILD patients in this study). We hope deep research into such anti-inflammatory pathways and regulatory mechanisms of inflammation might provide insights into understanding the biological basis of persistent pSS-ILD.

## Materials and methods

### Data collection and processing

**FIGURE 1:**
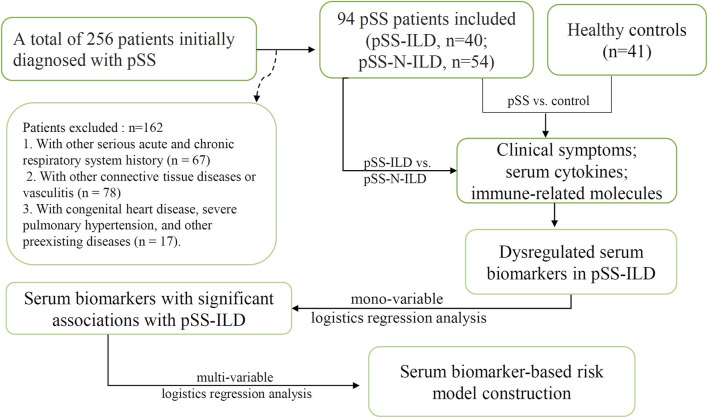
The flowchart of this study.

A total of 256 patients initially diagnosed with pSS in Henan Provincial People’s Hospital (Zhengzhou, China) from January 2019 to December 2022 were included. The work involving the serum specimens was reviewed and approved by the Ethics Committee of Henan People’s Hospital (approval number: 2020NL-09404). Informed consent was obtained from all the participants, and all the experiments were performed in accordance with relevant guidelines and regulations. Included patients met the 2002 American-European Consensus Group (AECG) criteria for pSS or the 2016 American College of Rheumatology (ACR)/European Alliance of Associations for Rheumatology (EULAR) classification criteria (Vitali et al., 2002; Shiboski et al., 2017). The exclusion criteria were as follows: 1) secondary dry syndrome or combined with other connective tissue diseases, such as arthritis, systemic lupus erythematosus, mixed connective tissue disease, vasculitis, polymyositis, or dermatomyositis (n = 78); 2) combined pulmonary infections, chronic obstructive pulmonary disease, pneumoconiosis, and lung diseases such as tumors, tuberculosis, and pulmonary nodules (n = 67); 3) patients with severe primary diseases of the heart, lungs, liver, kidneys, and other organs (n = 17). HRCT images were obtained from all patients at enrollment. The pSS patients whose imaging features supporting ILD in HRCT (e.g., ground-glass opacity, reticulation, consolidation, nodules, traction bronchiectasis, or honeycombing) were diagnosed as having pSS-ILD (Lohrmann et al., 2004). All enrolled patients were diagnosed for the first time and had not undergone treatment. Serum samples were obtained at the time of clinical data collection for all enrolled patients and stored at −80°C. The resulting cohort included 94 patients for the final analyses (Figure 1). The pSS patients were divided into a pSS-ILD group and a pSS-N-ILD group. A total of 54 pSS-N-ILD patients, aged 47.91 ± 9.97 years, and 40 patients with pSS-ILD, aged 60.1 ± 6.74, who were hospitalized in the Rheumatology and Immunology Department and Nephrology Department of Henan People’s Hospital from January 2019 to December 2022, were included. As pSS mainly occurred in the female population, all the included patients were women. For the control group, 41 women (aged 49.66 ± 8.67) who underwent physical examination in the hospital were included.

### The clinical symptoms and the serological biomarkers

The clinical symptoms, including dry eyes, dry mouth, dry cough, dyspnea, and arthrosis symptoms, were obtained through the medical record review of the patients. The anti-CCP antibodies were determined by ELISA kits (Euroimmun AG, Lübeck, Germany). Indirect immunofluorescence assay (IIF) was used to detect antinuclear antibodies (ANAs). The IIF-ANA screening test was performed using HEp20-10/liver biochip (Euroimmun AG, Lübeck, Germany). Sera were considered positive for ANAs if IIF staining was observed at a serum dilution of 1:100, and patterns were evaluated as semi-quantitatively 1+ to 4+ according to the intensity of the positive control. IIF patterns were determined according to an expert’s consensus on the clinical application of antinuclear antibody detection (PREPARE-2023CN385). The anti-Ro52, anti-SSA, and anti-SSB levels were detected with a line immunoassay (LIA) by EUROLINE kits (Euroimmun AG, Lübeck, Germany). The C-reactive protein (CRP), immunoglobulins (IgG, IgM, and IgA), and complements C3 and C4 were detected with an immunoturbidimetry assay (Roche Cobas6000 E501). The cytokines (IL-1β, IL-2, IL-4, IL-5, IL-6, IL-10, TNFα, IFNγ, IL-12p70, and IL-17) were detected by flow immunofluorescence assay (Qingdao Raisecare Biotechnology Co.). All the biomarkers were detected according to the manufacturer’s instructions. The sera of the patients and controls were collected and stored at −80°C before detection.

### Statistical analysis

R 4.3.1 software was used for data analyses and visualization. Wilcoxon test was used to compare different groups. Chi-square test was used to compare the qualitative variables. Spearman’s correlation analysis was used to investigate the potential associations between the levels of the variables. For all the analyses, *p* < 0.05 was considered significant. Mono-variable logistics regression analysis was applied to the serum biomarkers with significant differences between pSS-ILD and pSS-N-ILD groups to investigate their associations with pSS-ILD. The variables with *p* < 0.001 were then applied to multi-variable logistics regression analysis to construct a risk model for pSS-ILD. A nomogram of the results of the logistics regression model was drawn to estimate and visualize the probability of the cases to be pSS-ILD.

## Results

### The clinical and demographic characteristics of pSS-ILD and pSS-N-ILD cases and the healthy controls

**FIGURE 2:**
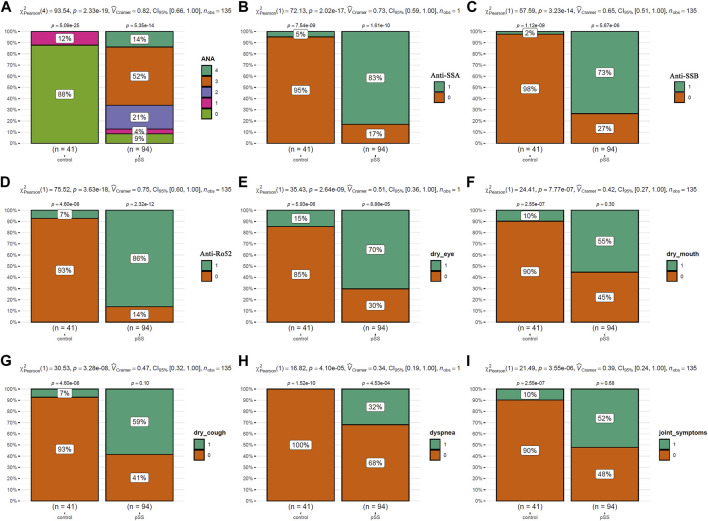
The comparisons of the autoantibody levels and the symptom occurrences between pSS cases and the healthy controls. **(A,B)** The comparisons of ANA, SSA, SSB, and Ro52 autoantibody levels between pSS cases and the healthy controls. **(E–I)** The comparisons of the occurrence of dry eye, dry mouth, dry cough, dyspnea, and arthrosis symptoms between pSS cases and the healthy controls. The chi-square test was used for comparisons, and *p* < 0.05 was considered significant. For **(A)**, 0–4 indicated negative, low, moderate, strong, and very strong levels of ANA antibodies in the serum. For **(B–I)**, 0 and 1 indicated negative and positive occurrences, respectively.

**FIGURE 3:**
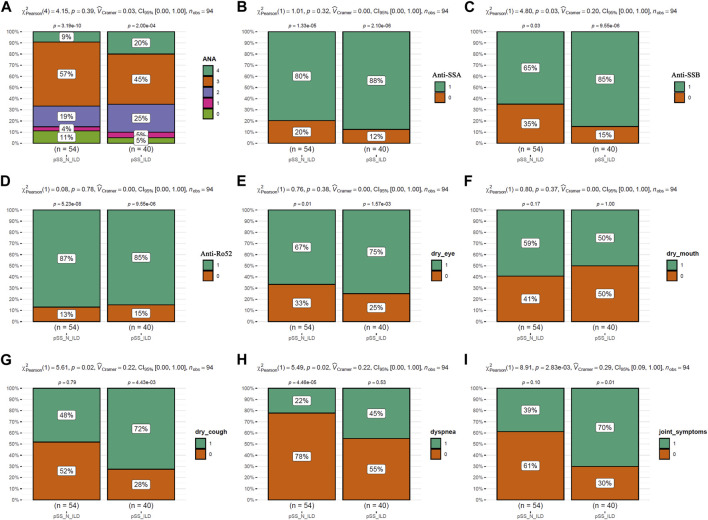
The comparisons of the autoantibody levels and the symptom occurrences between pSS subgroups. **(A,B)** The comparisons of ANA, SSA, SSB, and Ro52 autoantibody levels between the pSS-ILD and pSS-N-ILD groups. **(E–I)** The comparisons of occurrence of dry eye, dry mouth, dry cough, dyspnea, and arthrosis symptom occurrences between the pSS-ILD and pSS-N-ILD groups. The chi-square test was used for comparisons, and *p* < 0.05 was considered significant. For **(A)**, 0–4 indicated negative, low, moderate, strong, and very strong ANA antibodies in the serum, respectively. For **(B–I)**, 0 and 1 indicate negative and positive occurrences, respectively.

The clinical characteristics of the patients were presented in Table 1. As shown in Figure 2, the incidences of clinical symptoms, including dry eyes, dry mouth, dry cough, dyspnea, and joint symptoms, as well as the positive rate of autoantibodies, were significantly higher in pSS patients (pSS-ILD and pSS-N-ILD) than the healthy controls. No significant difference in anti-ANA, anti-SSA, anti-SSB, and anti-Ro52 titers and dry eye and dry mouth incidences was shown between the two pSS groups (Figure 3). Meanwhile, higher positive rates of anti-SSB and higher incidences of dry cough, dyspnea, and arthrosis symptoms were shown in the pSS-ILD patients than in the pSS-N-ILD patients.

**TABLE 1:** Clinical, demographic, and autoantibody characteristics of the pSS-ILD, pSS-N-ILD, and healthy control groups.

	Healthy controls	pSS-N-ILD	pSS-ILD
Demographic parameters	(n = 41)	(n = 54)	(n = 40)
Age	49.66 ± 8.67	47.91 ± 9.97	60.1 ± 6.74
Clinical characteristics			
Dry eyes	6 (14.6%)	36 (66.7%)	30 (75.0%)
Dry mouth	4 (9.8%)	32 (59.3%)	20 (50.0%)
Dry cough	3 (7.3%)	55 (58.5%)	26 (48.1%)
Dyspnea	0 (0)	12 (22.2%)	18 (45.0%)
Arthrosis symptoms	4 (9.8%)	21 (38.9%)	28 (70.0%)
Autoantibodies			
ANA	5 (2.2%)	48 (88.9%)	38 (95.0%)
Anti-SSA	2 (4.9%)	43 (79.6%)	35 (87.5%)
Anti-Ro52	3 (7.3%)	47 (87.0%)	34 (85.0%)
Anti-SSB	1 (2.4%)	35 (64.8%)	34 (85.0%)

### The dysregulations and heterogeneity of serum cytokines and immune-related molecules in pSS patients

**FIGURE 4:**
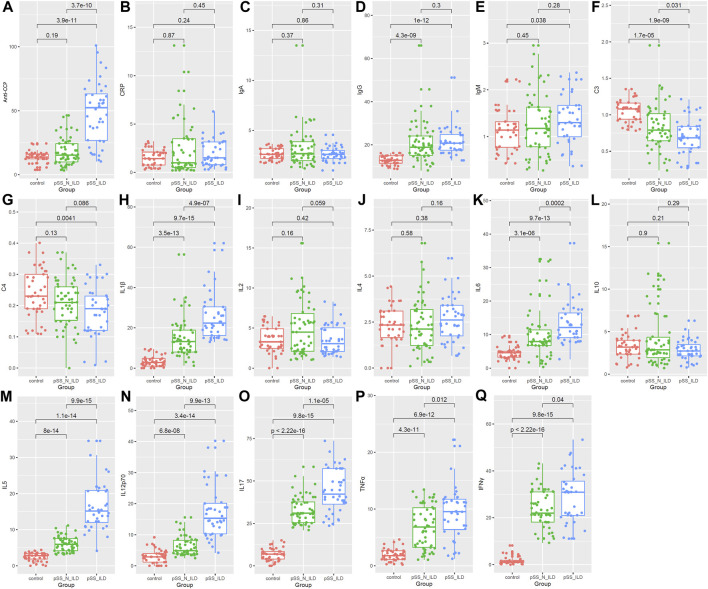
Comparison of serum biomarkers among the healthy group, the pSS-N-ILD group, and the pSS-ILD group. **(A,B)** anti-CCP and CRP comparisons between the healthy group, the pSS-N-ILD group, and the pSS-ILD group. **(C–E)** The IgA, IgG, and IgM comparisons between the healthy group, the pSS-N-ILD group, and the pSS-ILD group. **(F,G)** The complement (C3 and C4) comparisons between the healthy group, the pSS-N-ILD group, and the pSS-ILD group. **(H–O)** The comparisons of interleukin levels between the healthy group, the pSS-N-ILD group, and the pSS-ILD group. **(P,Q)** The comparisons of TNFɑ and INF-γ between the healthy group, the pSS-N-ILD group, and the pSS-ILD group. The Wilcoxon test was used, and *p* < 0.05 was considered significant.

As shown in Figure 4A, higher anti-CCP antibodies were present in pSS-ILD patients than pSS-N-ILD cases and healthy controls, while no significant difference between pSS-N-ILD cases and healthy controls was shown. There were no significant differences in CRP (Figure 4B) and IgA (Figure 4C) levels among the three groups. IgG levels were higher in pSS-ILD and pSS-N-ILD patients than the healthy controls. Moreover, no significant difference was shown between the two pSS subgroups (Figure 4D). Only pSS-ILD patients presented higher IgM levels than healthy controls, and there was no significant difference between the two pSS subgroups (Figure 4E). Lower levels of C3 and C4 (*p* < 0.05) were shown in pSS-ILD cases than in the healthy control group (*p* < 0.05) (Figures 4F, G), while only C3 presented a significant difference between the pSS-N-ILD cases and the two other groups (Figure 4F). Among the eight interleukins (Figures 4H–L), IL-1β (Figure 4H) and IL-6 (Figure 4K) were shown to be highest in pSS-ILD patients while lowest in the healthy controls. There were significant differences in the levels of IL-6 (Figure 4K), IL-5 (Figure 4M), IL-12p70 (Figure 4N), and IL-17 (Figure 4O) between each two of the groups, and they were highest in pSS-ILD patients while lowest in the healthy controls (*p* < 0.05). However, no significant differences of IL-2 (Figure 4I), IL-4 (Figure 4J), and IL-10 (Figure 4L) are shown. TNF-ɑ and IFN-γ also presented the highest levels in pSS patients and the lowest levels in healthy controls (Figures 4P, Q).

**FIGURE 5:**
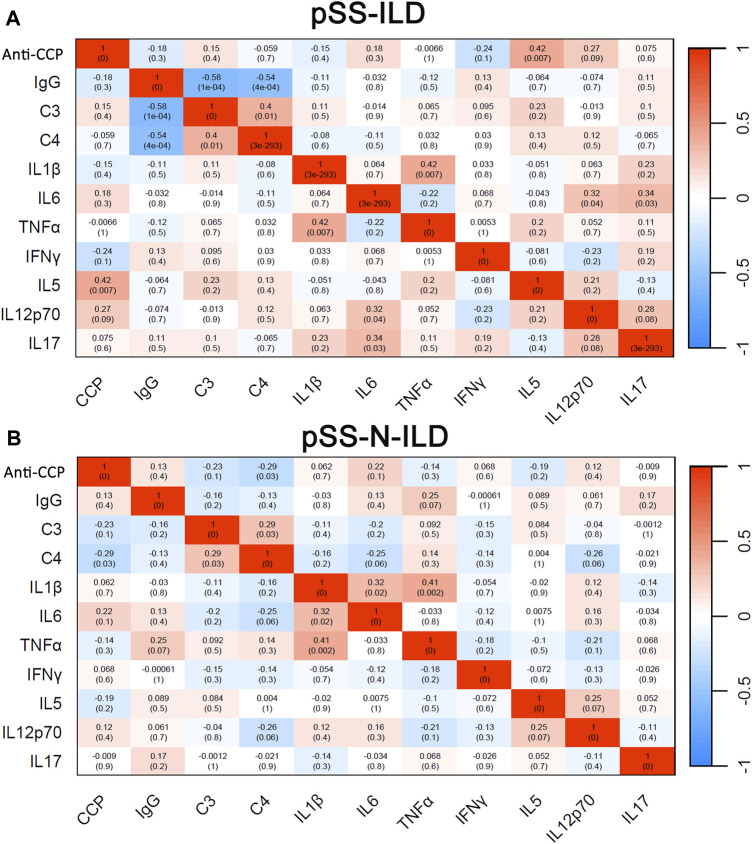
Binary correlation analysis of serum biomarkers in pSS patients. **(A,B)** Spearman’s correlations between the variables in pSS-ILD and pSS-N-ILD patients, respectively.

Through correlation analyses, the heterogeneity of the serum marker correlations in pSS patients with and without ILD was also shown. In contrast to the similar positive correlations between C3 and C4 and between IL-1ß and TNFɑ in pSS-ILD patients (Figure 5A) and pSS-N-ILD cases (Figure 5B), there were also significant differences in serum marker correlations between the two groups. The significant negative correlations of IgG with C3 and C4 (*r* = −0.58, *p* < 0.05, *r* = −0.54, *p < 0.05*) while the positive correlations of IL-12p70 (*r* = 0.32, *p* < 0.05) and IL-17 (*r = 0.34, p < 0.05*) with IL-6 were only shown in pSS-ILD patients (Figure 5A) but not pSS-N-ILD cases (Figure 5B). In contrast, the negative correlations of C4 with anti-CCP while the positive correlation IL-1ß with IL-6 in pSS-N-ILD patients (Figure 5B) disappeared in pSS-ILD patients (Figure 5A).

### The associations of the serum biomarkers with pSS-ILD

**FIGURE 6:**
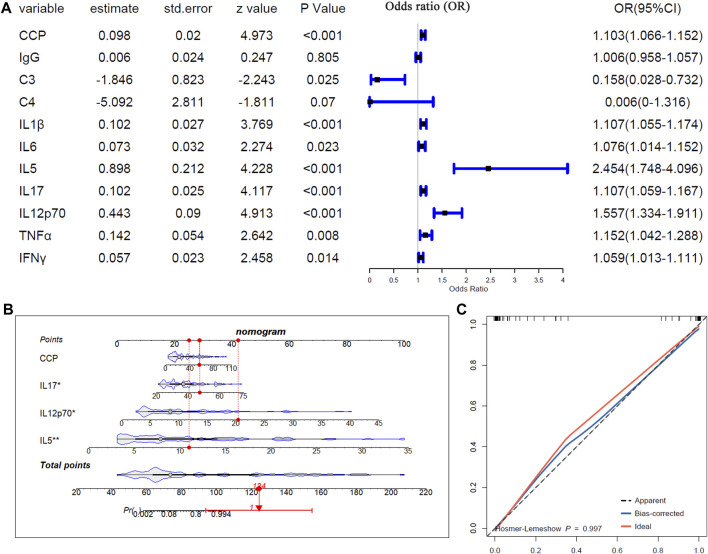
The associations of the serum biomarkers of patients with pSS-ILD. **(A)** Mono-variable logistic regression analysis of the serum biomarkers in pSS patients. **(B)** Multi-variable logistics regression analysis of the serum biomarkers in pSS patients. **(C)** The calibration curve of the multi-variable logistics regression model in pSS patients.

Logistics regression analysis was applied to study the relationships of the differentially expressed serum biomarkers, including anti-CCP antibody, IgG, C3, C4, IL-1ß, IL-5, IL-6, IL-17, IL-12p70, TNF-α, and INFγ), between pSS-N-ILD and pSS-ILD. As shown in Figure 6A, except IgG and C4, all the other serum biomarkers were shown to be associated with pSS-ILD. C3 was indicated a protective factor for the cases (OR <1, *p* < 0.05) and anti-CCP, IL-1ß, IL-5, IL-6, IL-17, IL-12p70, TNF-α, and INFγ were risk factors for pSS-ILD occurrence (OR >1, *p* < 0.05). The four variables (anti-CCP, IL-17, IL-12p70, and IL-5) with *p* < 0.001 in the mono-variable regression analysis were then applied to multi-variable analysis to identify independent risk factors for pSS-ILD. As shown in Figure 6B, IL-17, IL-12p70, and IL-5 were shown to be independent risk factors for pSS-ILD. The efficiency of the risk model was confirmed by the calibration curve in Figure 6C. The results indicated that the higher levels of IL-17, IL-12p70, and IL-5 indicated a higher risk of the cases being pSS-ILD.

## Discussion

Immune dysfunction, especially abnormal cytokine production, plays an important role in the pathogenesis of pSS. The lung is the most vulnerable organ outside the glands of pSS, with poor prognosis and high mortality (Gupta et al., 2019). Cytokines modulate the response of different cellular lineages and induce the secretion of more critical cytokines (Simon et al., 2021). Many studies showed that the persistent presence of chronic inflammatory cytokines is one of the main factors contributing to the development of pSS-ILD (Fogel et al., 2018; Ibrahem, 2019; Peredo and Beegle, 2021). The current clinical treatment for pSS mainly involves controlling inflammation and anti-fibrosis strategies (Luppi et al., 2020b; van den Hoogen and van Laar, 2020). This study presented serious disorders of cytokines and humoral immunity in pSS-ILD patients, which indicated the important roles of abnormal immune response in pulmonary interstitial lesions. A risk model was constructed to predict the ILD status of pSS patients, and several cytokines were shown to be risk factors for pSS-ILD, providing new potential targets for its treatment.

The clinical symptoms of pSS patients vary because of the damage to various target organs. We found no significant difference in the clinical incidence of dry eye and dry mouth between the pSS-N-ILD cases and the pSS-ILD patients. Meanwhile, the incidence of dry cough, dyspnea, and joint symptoms in pSS-ILD was significantly higher than that in pSS-N-ILD. These results indicated that the common biomarkers and inflammatory signaling pathways were involved in joint and lung damage of pSS-ILD patients. The level of anti-CCP was much higher in the pSS-ILD group than in the pSS-N-ILD group. Moreover, anti-CCP was a marker of the diagnosis of rheumatoid arthritis (Yang et al., 2019). It was reported that the elevated CCP antibody level could increase the incidence of RA-ILD, and the CCP antibody was found in sputum samples of RA patients with a risk of ILD (Correia et al., 2019). It suggested that lung tissue might be one of the sources of target antigens for CCP antibodies.

The levels of some inflammatory cytokines were also strongly associated with the development of pSS-ILD. Research confirmed that Th2 cells can cause a profibrotic effect in IPF and pulmonary nodular lesions (Kolahian et al., 2016). IL-5, which is one of the Th2 cytokines, induces proliferation and differentiation of B cells (Li et al., 2017). In this study, we found that the level of IL-5 was significantly higher in the pSS-ILD group than in the pSS-N-ILD group. There was a statistically significant positive correlation between IL-5 and anti-CCP in pSS-ILD, but not in pSS-N-ILD, indicating the differences and complexity of the cytokine network during pSS progression. Noguchi et al. found that IL-6 receptor monoclonal antibodies (tocilizumab) can alter the ratio of circulating effector B lymphocytes and naïve B cells, reduce the level of CCP antibodies, and alleviate clinical manifestations in RA patients (Noguchi et al., 2020). These results reflect that the inflammatory signaling pathway of IL-6 may promote the production of CCP antibodies. Here, we also found that the levels of IL-6, IL-12p70, and IL-17 were overexpressed in pSS-ILD patients. Positive correlations between IL-6 and IL-12p70 with IL-17 were shown in pSS-ILD but not in pSS-N-ILD, indicating the complexity of the cytokine network. Previous studies have shown that IL-6 and IL-12p70 secreted by DCs induce naïve CD4 + T cells to Th17 and Th1 differentiation, which promotes the expression of IL-17 (Zhuang et al., 2023). IL-6, IL-12p70, and IL-17 could interact with each other and commonly participate in the development of ILD and joint symptoms by activated B lymphocytes and induced naïve CD4^+^ T cells in pSS-ILD patients. Therefore, IL-5, IL-6, IL-12p70, IL-17, and CCP antibodies may be associated with higher risk of ILD.

Multiple cytokines are expressed at high levels in the pSS-ILD group and participate in the deposition of the extracellular matrix and the differentiation of fibroblasts by multiple pathways, leading to the occurrence of pulmonary interstitial lesions. No single cytokine can explain complex immune signaling pathways and all clinical symptoms. Here, we found positive correlations among multiple cytokines. Therefore, analyzing a group of (Th1/Th2) cytokines can more accurately identify high-risk factors for pSS-ILD disease. In this study, multi-variable logistics regression complemented the findings that IL-17, IL-12p70, and IL-5 were independent risk factors for pSS-ILD. The higher levels of IL-17, IL-12p70, and IL-5 indicated a higher risk of the cases to be pSS-ILD.

We also revealed significant differences in positive rates of antibodies between pSS subgroups. In recent studies, significantly elevated IgG levels were found in pSS-ILD patients (Zhang et al., 2020). A similar trend of IgG levels was found in pSS-N-ILD and pSS-ILD patients. The level of IgG and the positive rate of ANAs and anti-SSA/Ro52 were not significantly different between pSS subgroups. However, the anti-SSB positivity was higher in the pSS-ILD group. Previous studies reported that the isolated anti-SSB was associated with complications or “high-risk” phenotype patients (Parisis et al., 2020). In addition, anti-SSA/SSB antibodies have long been considered pivotal markers in diagnosing pSS (Shiboski et al., 2012).

Our study had some limitations. First, as only female patients were included in this study, the application of the results to male patients was limited. Second, the sample size was limited, and all the patients were Chinese. A larger cohort validation of the results was needed in further studies. Third, evaluating other parameters, such as activity and damage score, might improve the power of the risk model. These parameters could be evaluated in future analyses.

## Conclusion

In summary, this study revealed significant differences of symptom positive rates and serum biomarker levels between pSS-ILD patients and pSS-N-ILD cases. The positive associations of IL-17, IL-12p70, and IL-5 with pSS-ILD were indicated through mono-variable and multi-variable logistics regression analyses. The higher expressed cytokines might be key indicators for the occurrence of pSS-ILD and pSS progression. The clinical and serological heterogeneity between pSS patients with and without ILD might be influenced by the cytokine microenvironment. Blocking the signaling pathways of these cytokines may be a potential target for controlling progression of pSS-ILD.

## Data Availability

The original contributions presented in the study are included in the article; further inquiries can be directed to the corresponding authors.
